# Neurostimulation

**DOI:** 10.1155/2016/1830405

**Published:** 2016-09-22

**Authors:** Helena Knotkova, Michael A. Nitsche, Volker Tronnier

**Affiliations:** ^1^MJHS Institute for Innovation in Palliative Care, New York, NY, USA; ^2^Department of Family and Social Medicine, Albert Einstein College of Medicine, The Bronx, NY, USA; ^3^Department of Neurology, University Medical Hospital Bergmannsheil, 44789 Bochum, Germany; ^4^Department of Psychology and Neurosciences, Leibniz Research Centre for Working Environment and Human Factors, 44139 Dortmund, Germany; ^5^Department of of Neurosurgery, Universitätsklinikum Schleswig-Holstein, Campus Lübeck, Ratzeburger Allee 160, 23538 Lübeck, Germany

Neurostimulation encompasses a broad range of invasive and noninvasive techniques that aim for enduring alterations of neuronal activity, or excitability. This bears enormous clinical potential, because pathological changes in neural activity are common in many diseases and neurostimulation techniques can be employed to attempt functional normalization of the neural circuitry. Further, neurostimulation techniques can facilitate insight into neurophysiological mechanisms underlying the development and maintenance of difficult-to-treat disorders and symptoms, as well as provide insight into a linkage between neurophysiological characteristics of neural networks and changes in functional and behavioral outcomes.

The field of neurostimulation has enormously expanded over the past decades. Technological progress in both biomedical sciences and neurostimulation industry facilitated advances in understanding neural changes in the central nervous system that represent functional targets for neurostimulation and mechanisms that underlie the neurostimulation effects. Hand in hand with growing knowledge, new open questions and challenges have emerged, facilitating further technological progress, translational research, and clinical applications in this cutting-edge biomedical field.

This special issue brings an exciting array of original reports and reviews that examine putative mechanisms and neurophysiological effects of invasive and noninvasive neurostimulation techniques, such as transcranial magnetic stimulation, transcranial direct or alternating current stimulation, random noise stimulation, or motor cortex stimulation. Overall, this special issue advances the existing evidence on growing potential of neurostimulation in modern medicine and biomedical sciences.

*Helena Knotkova* *Helena Knotkova* *Michael A. Nitsche* *Michael A. Nitsche* *Volker Tronnier* *Volker Tronnier*

